# Effect of Friction Stir Process Parameters on the Mechanical and Thermal Behavior of 5754-H111 Aluminum Plates

**DOI:** 10.3390/ma9030122

**Published:** 2016-02-23

**Authors:** Livia Maria Serio, Davide Palumbo, Luigi Alberto Ciro De Filippis, Umberto Galietti, Antonio Domenico Ludovico

**Affiliations:** Politecnico di Bari, Department of Mechanics Mathematics and Management (DMMM), Viale Japigia 182, Bari 70126, Italy; liviamaria.serio@poliba.it (L.M.S.); luigi.defilippis@poliba.it (L.A.C.D.F.); umberto.galietti@poliba.it (U.G.); antoniodomenico.ludovico@poliba.it (A.D.L.)

**Keywords:** friction stir welding (FSW), aluminum alloy (AA), ultimate tensile strength (UTS), infrared thermography (IRT)

## Abstract

A study of the Friction Stir Welding (FSW) process was carried out in order to evaluate the influence of process parameters on the mechanical properties of aluminum plates (AA5754-H111). The process was monitored during each test by means of infrared cameras in order to correlate temperature information with eventual changes of the mechanical properties of joints. In particular, two process parameters were considered for tests: the welding tool rotation speed and the welding tool traverse speed. The quality of joints was evaluated by means of destructive and non-destructive tests. In this regard, the presence of defects and the ultimate tensile strength (UTS) were investigated for each combination of the process parameters. A statistical analysis was carried out to assess the correlation between the thermal behavior of joints and the process parameters, also proving the capability of Infrared Thermography for on-line monitoring of the quality of joints.

## 1. Introduction

The process of Friction Stir Welding (FSW) is a solid state welding method based on frictional and stirring phenomena, which was discovered and patented by the Welding Institute of Cambridge in 1991 and documented in the literature by Thomas [1], Nandau *et al.* [2], and Rodrigues *et al.* [3].

In this process, welding heat is produced by a rotating, non-consumable tool, which plunges into the work piece and moves forward. Therefore, realization of welds is possible, thanks to the action of a tool that generates heat from friction between its shoulder and the base material, giving rise to plastic deformation with its pin. Significant advantages can be obtained when compared with fusion joining processes for aluminum due to a very low welding temperature: mechanical distortion is practically eliminated, with minimal Heat Affected Zone (HAZ), and there is an excellent surface finish [2].

In the literature, there are numerous contributions regarding the application of this process, which is used with success both for welding of low-melting temperature alloys and for steel plates with considerable thickness and also for dissimilar materials which are generally considered difficult for fusion-welding and for which large forces are needed. Dressler *et al*. [4] studied the application of the Friction Stir Welding process to join the titanium alloy TiAl6V4 to aluminum alloy AA2024-T3; in the work of Aonuma *et al.* [5], the effects of alloying elements on interface microstructure of Mg-Al-Zn magnesium alloys and titanium were analyzed, while Kostka *et al.* [6] studied the microstructure of FSW joints of aluminum alloy to magnesium alloy.

The FSW process of non-heat-treatable aluminum-magnesium (Al-Mg) alloys (5XXX series), is substantially less explored in the literature despite the huge interest for the AA 5XXX alloys in automotive construction. In particular, the strong influence of process parameters on the quality of aluminum joints has been demonstrated in terms of tensile strength, fatigue behavior and residual stress.

The mechanical properties of the welds produced from AA5XXX alloys depend mainly on the grain size and the dislocation density due to the phenomena of plastic deformation and recrystallization occurring during the FSW process, as shown by Senkara *et al.* [7] and by Miles *et al.* [8].

Peel *et al.* [9] analyzed the microstructure, the mechanical properties and the residual stresses of Friction Stir Welds of aluminum alloy AA5083 while Kwon *et al.* [10], studied the application of Friction Stir Welding to 5052 aluminum alloy plates.

Jin *et al.* [11] studied the Friction Stir Welding of Al5754 alloys using constant FSW parameters and they have also examined the microstructural development and micro hardness distribution in the welds. Attallah *et al.* [12] conducted a study on the FSW of 2XXX and 5XXX series sheet materials in various tempers at different FSW parameters. These analyses have highlighted the relationship between the banding of constituent particles and the “onion rings” formation in the Al5754 joint.

Casalino *et al.* [13] documented a previous partial experiment on the 5754 aluminum alloy and used a hybrid system for combining the laser welding with the FSW process.

In the work of Kulekci *et al.* [14], the effects of the tool pin diameter and tool rotation speed at a constant traverse speed, were investigated on fatigue properties of friction stir overlap welded Alloy Al5754. Two other works [15,16] provide information on the influence of process parameters, on the tensile and the fatigue behavior of a Friction Stir Welded joint under a single FSW parameter in a tailor-welded blank of Alloy Al5754. These studies, however, have not been explicit with regard to the process parameters that were employed.

In the above mentioned works, mechanical tests and destructive tests are used to evaluate the quality of joints such as micrographs and macrographs or X-ray diffraction used to measure residual stress [9,10,11,12,13,14,15,16,17] These techniques cannot provide information about the performance of process during welding and require lengthy test times, making them unfeasible for the industrial field.

Other authors propose Infrared Thermography (IRT) to study the thermal behavior of welded joints. IRT is a full-field contactless technique used for non-destructive evaluation of defects in a wide range of materials and for process monitoring [18,19,20,21,22] In particular, in the work of Palumbo *et al.* [23,24], the fatigue behavior of steel welded joints was studied by means of thermal methods while automatic on-line defect detection of aluminum alloy plates welded by the TIG process was proposed in the work of Sreedhar *et al.* [25].

With regard to the FSW process, there are only a few studies on thermal monitoring. Hwang *et al.* [26] carried out an experimental study of temperature distributions within the work piece during Friction Stir Welding of aluminum alloys while Zhu *et al.* [27] performed a numerical simulation of transient temperature and residual stresses in FSW process of 304L stainless steel; Chao *et al.* [28] assessed the heat transfer in Friction Stir Welding both experimentally and numerically, and Schmidt *et al.* [29] developed an analytical model for heat generation in FSW.

All these works show applications based only on the measurement of absolute temperature of welded joints during the process with infrared cameras or thermocouples. The works of Serio *et al.* [17,18] demonstrate how the absolute temperature is affected by environmental conditions and is influenced by experimental set-up adopted for the tests; subsequently it cannot be used as a representative parameter of the FSW process. In particular, a more sensitive thermal parameter is proposed for the monitoring of the FSW process, representing the heat generated during the process [18,30].

The aim of this work is to develop an efficient procedure for monitoring and assessing the quality of 6 mm thick sheets of 5754-H111 aluminum alloy welded by FSW process. The quality of welded joints is defined in terms of mechanical properties and macroscopic defects produced during the process, and it is correlated to the thermal parameters monitored during FSW process with IRT.

## 2. Materials and Methods

### 2.1. Data Analysis and Empirical Models Used in the Work

In this work, a number of models were used to study the FSW process. In particular, the quality of welded joints was correlated to the process parameters (FSWP) in three ways.

A qualitative analysis was performed with non-destructive (visual inspection) and destructive testing (macrographic tests) in order to detect macro defects present on the surface and within the welded area.

**Figure 1:**
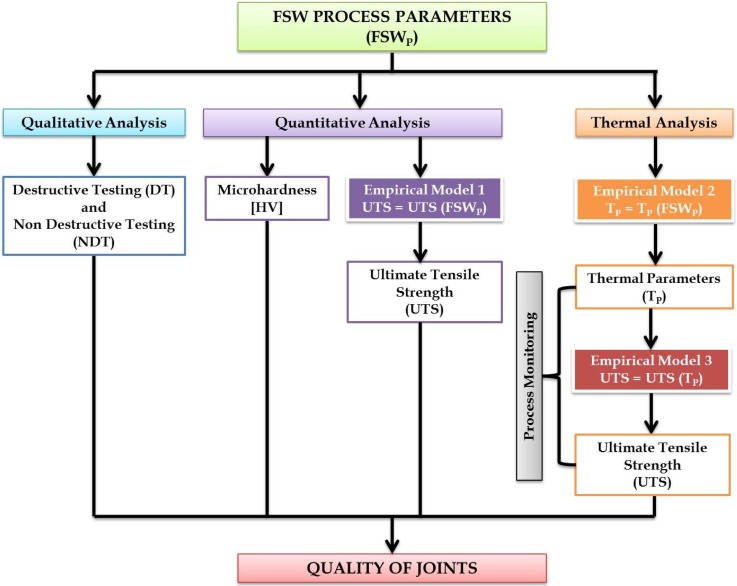
Empirical models used for evaluating the quality of FSW process by means of destructive and non-destructive tests.

The ultimate tensile strength (UTS) of joints was considered in order to perform a quantitative analysis of process and an empirical model was developed (Model 1, see Figure 1) to describe the influence of process parameters on the quality of joints. Moreover, Vickers microhardness tests were carried out on all cross-sections perpendicularly to the welding direction and in the mid-section of all samples.

Thermal behavior of FSW process was investigated by means of thermographic technique. In particular, two thermal parameters (*T*_p_) were considered: the maximum temperature and the slope of the heating curve measured during the FSW process [17,18]. The last parameter was correlated with the process parameters through a second empirical model (Model 2, see Figure 1).

Finally, the quality of welded joints, in terms of UTS, was directly correlated to the thermal parameters with a third model (Model 3, Figure 1). This model allows for monitoring of the FSW process in a quantitative manner, knowing the thermal behavior of joints during the process.

### 2.2. Materials and Welding Parameters

The investigation was carried out on plates of 5754-H111 aluminum-magnesium alloy with 6 mm thickness, obtained according to the rolling direction with the following dimensions: Length × Width × Height = 200 mm × 100 mm × 6 mm. This aluminum alloy is characterized by excellent corrosion resistance in the marine environment and it presents high formability, so this alloy is of great application both in the automotive and naval fields.

The chemical compositions and principal mechanical properties of the AA5754-H111 alloy are, respectively, presented in Table 1 and Table 2.

All welds were carried out in position control using LEGIO FRICTION STIR WELDING (ESAB, Sweden) which was equipped with the FSW welding head.

**Figure 2:**
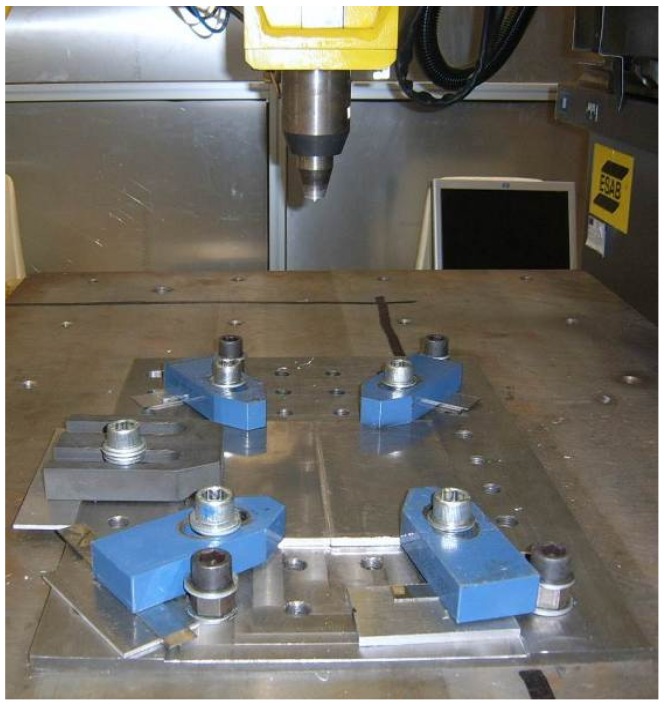
Positioning of the work piece on the fixture table.

The work piece was fixed on a rigid backing-plate, and clamped along the welding direction on both sides to avoid lateral movement during welding. The terminal part of the work piece was positioned on the worktables, as shown in Figure 2.

The welding direction was chosen parallel to the rolling direction and the dwell time was always kept at 15 s, whereupon the tool was moving with constant traverse speed according to the parameters combination selected and described below. During the penetration phase, the rotating tool pin penetrates into the work piece until the tool shoulder makes contact. The penetration speed is about 0.5 cm/min, the dwell time is 15 s. The tool has a diameter of the shoulder of 22 mm, a height of the pin of 5.8 mm and a tilt angle of 1.2° to facilitate the mixing of the material.

**Figure 3:**
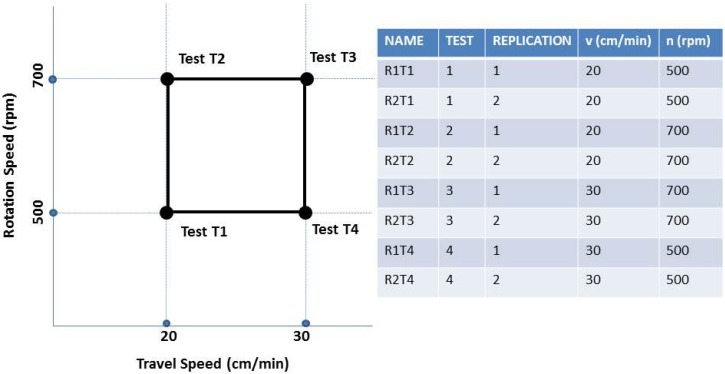
Full Factorial Plane 2^2^.

The considered values of tool rotation speed and the traverse speed were respectively: 500, 700 RPM and 20, 30 cm/min. These process parameters were chosen as a basis for the construction of the first experimental plan (Figure 3). In particular, a 22 full factorial experimental plan considers 4 combinations of the 2 parameters and 2 replications for each combination.

The choice of the process parameters is derived from a previous work [31] in which is documented a partial experiment of FSW on 5754 aluminum alloy. To our knowledge, reference [31] is the only work on this aluminum alloy with plates of 6 mm.

Each test in the following sections has been denominated in accordance with the parameters combination of tool rotation speed n and traverse speed v. For example, the indication “R1T1”, refers to Replication 1 and Test 1.

In correspondence with each parameter combination, a characteristic parameter was calculated, called “Weld Pitch” (mm) that indicated the ratio between the traverse speed v and the tool rotation speed n. The importance of this parameter was documented by Nandan *et al.* [2].

This parameter was closely related to the specific heat input given to the joint during the process, and it could represent an indication of the welding quality.

Specifically, previous studies have identified for the alloys of the 5XXX series a welding optimal pitch of 0.35 mm. Thus, from this indication, it is estimated, already in this step, that the parameter configuration able to obtain an optimal weld pitch is Test 1 (*v* = 20 cm/min − *n* = 500 RPM − WP = 0.4 mm).

### 2.3. Experimental Set-up

#### 2.3.1. Visual Inspections

Visual inspections and macrographic tests have been carried out preparing the cross-sectional samples taken from all welded joints. Specimens were prepared using standard metallographic methods for macroscopic examinations of the weld zones.

The face examination of the welds was carried out according to the criterion fixed by the Standard UNI EN ISO 25239:2011 [32].

Cross sections of the welds were cold mounted, polished and etched with a solution consisting of 5 mL of distilled water and 120 mL of hydrochloric acid for 90 s.

After these treatments, the samples were observed with a high resolution digital camera (Mod.: Canon EOS 40D, 10 MPixel) to detect large and very small internal flaws.

#### 2.3.2. Microhardness

**Figure 4:**
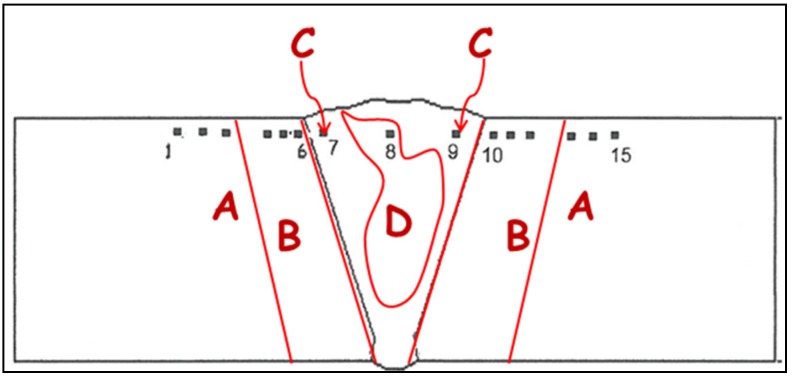
The measurement of Vickers microhardness for the FSW joint: A: Unaffected material; B: Heat affected zone (HAZ); C: Thermo-mechanically affected zone (TMAZ); and D: Weld nugget (Part of thermo-mechanically affected zone).

The Vickers microhardness of the weld zone was measured on all cross-sections perpendicularly to the welding direction and in the mid-section of all the samples using a Vickers indenter Remet (REMET, Bologna, Italy) HX 1000 50 gf load for 15 s. A total of 15 indentations were performed in each measurement of the hardness profiles of the samples. Thus, the Vickers microhardness of the characteristic friction stir weld zone was measured in all points indicated in Figure 4 using a Vickers indenter.

#### 2.3.3. Thermal Analysis

The surface thermal acquisitions were performed using two thermocameras. In particular, in order to acquire thermal data along the weld tool direction, the cooled FLIR X6540 SC IR camera (FLIR System, Inc., Wilsonville, OR, USA) was used, which latter has thermal sensitivity (NETD) < 20 mK and is based on a InSb photonic detector with 640 × 512 pixels. The uncooled FLIR SC640 IR camera (FLIR System, Inc., Wilsonville, OR, USA) was placed in a perpendicular direction with respect to the first thermocamera (thermal sensitivity (NETD) < 30 mK, 640 × 480 pixels). Sequences were recorded during each test captured at 15 Hz.

**Figure 5:**
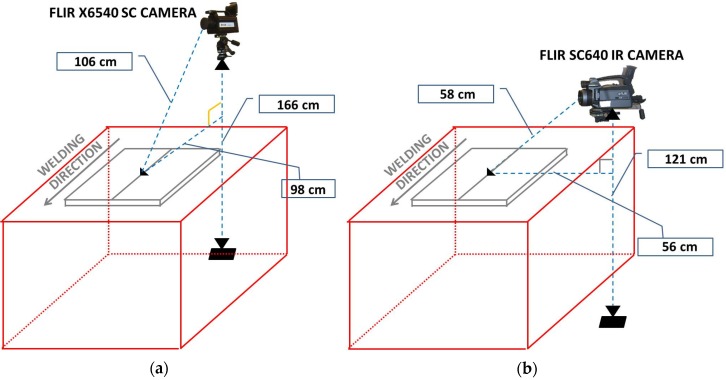
Set-up used for thermographic data acquisition: (**a**) FLIR X6540 sc IR camera placed along the welding tool direction; and (**b**) Flir sc 640 IR camera placed perpendicular to the welding tool direction.

Both thermocameras recorded the maps of surface temperature, across the weld, for each combination of process parameters in accordance with the experimental plan described and shown in Figure 3. The thermographic set-up is shown in Figure 5.

Before the tests, specimens were painted with matt black paint (Figure 5b) to uniform the emissivity of treated surfaces to 0.95 and to avoid reflections caused by heat sources placed near the specimens during the tests.

#### 2.3.4. Tensile Tests

**Figure 6:**
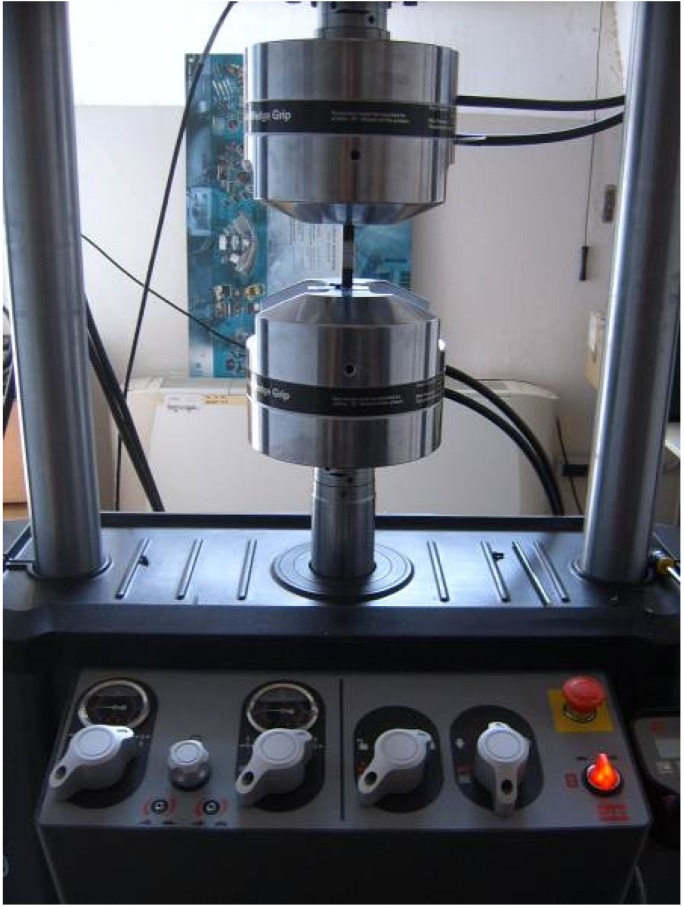
Setup of tensile test.

Ultimate tensile strength (UTS) was used to evaluate the mechanical properties of welded joints. All tests were performed on a MTS servo hydraulic machine (Model 370, MTS System Corporation, Eden Praire, MN, USA, see Figure 6), under displacement control with a constant crosshead speed displacement rate of 5 mm/min according to Standard UNI EN ISO 6892-1:2009 [33].

## 3. Results and Discussion

### 3.1. Visual Inspection and Macrographs

All sheets were visually inspected after welding. Generally speaking, qualitative inspection of the welds was performed by visual examination to detect surface defects, followed by metallographic analysis to detect internal flaws (reported in the following section).

Interesting results can be obtained by visual inspection because of the possibility of verifying the presence of possible macroscopic external defects, such as surface irregularities, excessive flash [10], and lack of penetration or surface-open tunnels.

Typically, the surface appearance of FSW is a regular series of partially circular ripples, which pointed towards the start of the weld. It was observed that these ripples were essentially cycloidal and were produced by the final sweep of the trailing circumferential edge of the shoulder during traverse.

The rotation speed of the tool and traverse speed of the work piece determines the pitch between the ripples. It was observed that with the same rotation speed, by increasing the traverse speed, an increase of the roughness of the joints surface occurred.

**Figure 7:**
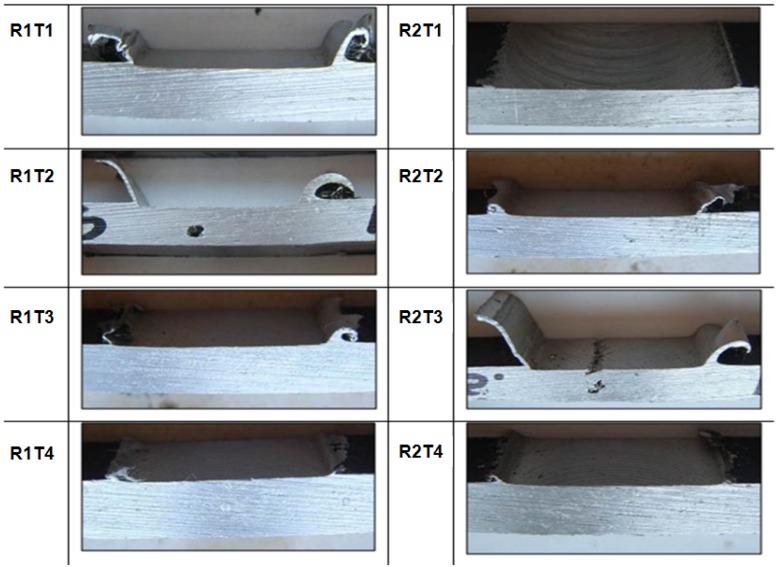
Visual examination of the section of joints.

**Figure 8:**
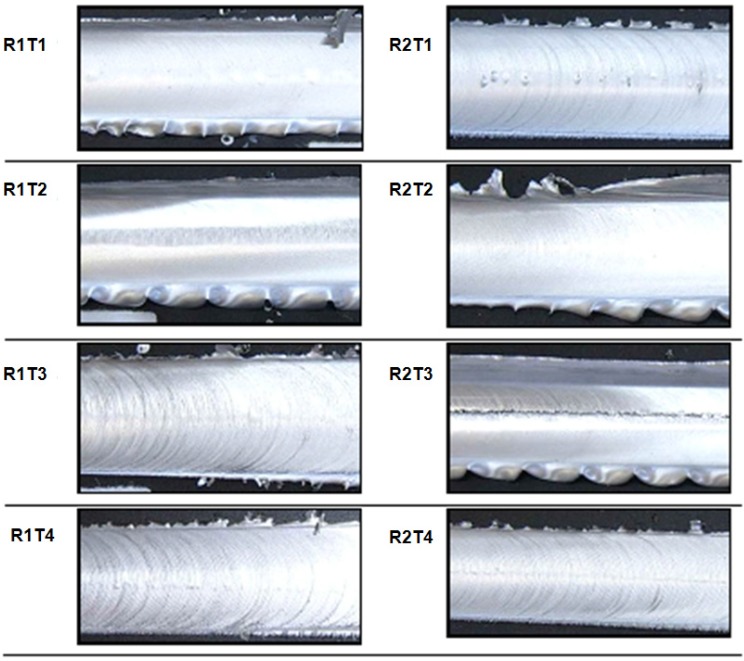
Visual examination of the surface of joints.

All the performed observations on welded specimens along the cross sections (perpendicular to the welding direction) and the junction line, are shown in Figure 7 and in Figure 8, respectively.

The qualitative inspection of the welds allows for observation of any defects and surface characteristics, in particular:

- For all the tests performed, the surface appearance of FSW showed a regular series of partially circular ripples. These ripples are essentially cycloidal and are produced by the circumferential edge of the shoulder during traverse. The rotation speed of the tool and traverse speed of work piece determines the pitch between the ripples.
- Some tests (R2T3, R1T2, R2T2) were characterized by an excessive presence of lateral flash, resulting from the outflow of plasticized material from underneath the shoulder [10].
- The other tests showed continuous flash but with a marked ripple. This demonstrated the significant ductility of the material, and with the plastic deformation suffered by the material, changes periodically over time.

In Tests 2 and 3, characterized by a tool rotation of 700 RPM, macro voids, denominated “tunnel”, were present in the section. In Test R2T3, the voids also occurred on the surface of the weld, creating a groove along the length of the weld. The cause of these defects could be due to the use of incorrect process parameters, which provided wrong heat input per unit length impeding a correct mixing action of the material, creating voids in the section and on the surface of the welding [31].

Table 3 summarizes the results of the visual analysis and the weld pitch is highlighted because it was considered an indicator of good welding quality. Based on the reference weld pitch (0.35 mm), Test 1 (*n* = 500 rpm, *v* = 20 cm/min) was the one nearest to the optimal ratio (WP = 0.40 mm). For this test, no defect was detected, both on the surface and in the section.

Visual inspection on Test 4 provided good results, also thanks to an excellent surface finish of the welded joints, but the corresponding value of weld pitch moved away from the reference (0.60 *vs.* 0.35).

Macrographic analyses were carried out to detect internal flaws of the welds.

**Figure 9:**
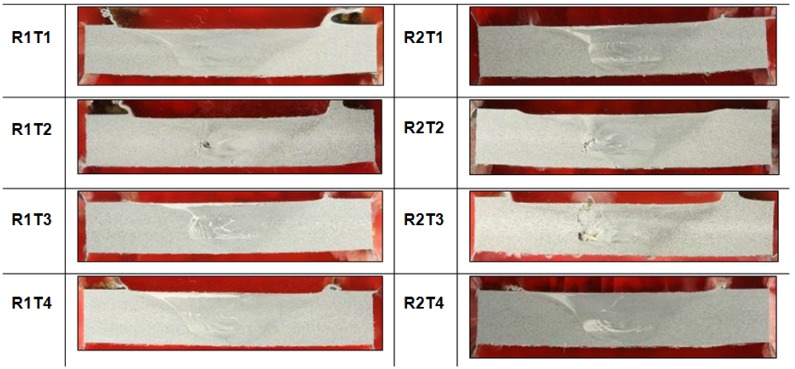
Macrographs of the cross section of the welds for the different welding parameters.

All macrographic examinations (Figure 9) clearly displayed the structure of all joints. Almost all analyses revealed a good mixing and a good penetration of the tool in the joints, except for the joints section realized using the highest rotation speed (*n* = 700 RPM; tests: R2T3, R1T2, and R2T2), where defects such as cavity, due to inappropriate contribution of heat input and stirring rate have been observed. These results were in agreement with the ones observed in the visual inspection.

All macrographs presented a nugget shape, not well defined with a notably elongated form, while the typical “onion rings” that identified the mixing zone characteristic of FSW process, were very visible [2,12].

### 3.2. Microhardness Measurement

**Figure 10:**
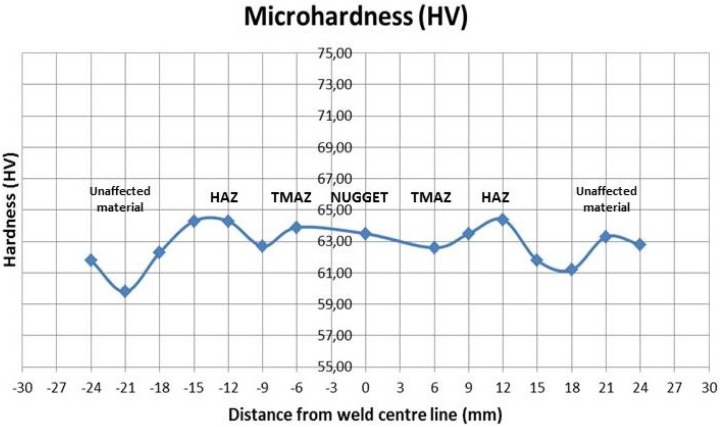
Microhardness distribution of Test 4.

The effects of the FSW on the hardness distribution were fully analyzed. It was observed that the weld samples with the same traverse speed had similar profiles. Thus, for example in Figure 10, there is the hardness distribution of Test 4 and it shows the trend of Vickers microhardness typically tracked in friction stir joints. From all the results, no HAZ softening was found, which was expected given the fact that tempered H111 is almost equivalent to the O-temper, which is better specified in the work of Threadgill *et al.* [31].

In almost all tests, the highest value of microhardness in the stirred zone was found not in the middle of the joint, but shifted towards one side of the joint, where higher plastic strain was observed and the microhardness curve shows a W-shape.

The average hardness in the nugget and in the base material is similar among all samples.

The hardness profile greatly depends on the precipitate distribution and only slightly on the grain and dislocation structure [34]. Thus, the evolution of the precipitate distribution with the experienced temperature peak and with the stain introduced during the welding produces the observed hardness variation.

### 3.3. Thermal Behavior of Joints

**Figure 11:**
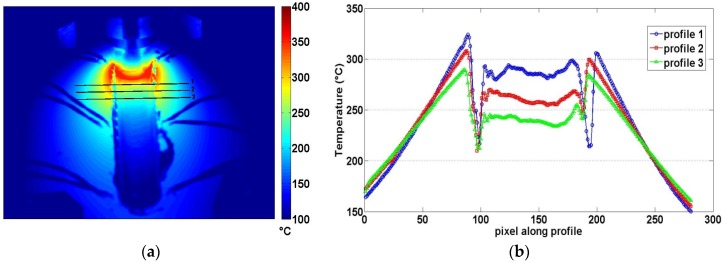
(**a**) Image obtained at the end of the welding; (**b**) Three thermal profiles acquired during Test 1 in a fixed time instant.

**Figure 12:**
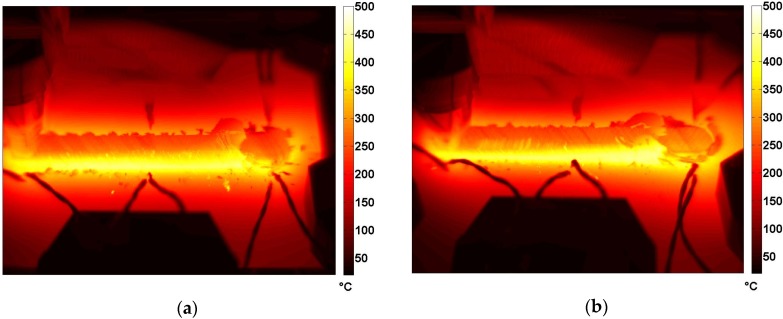
Maps of Tmax assessed for: (**a**) Test 1: *v* = 20 (cm/min) − *n* = 500 (RPM); and (**b**) Test 4: *v* = 30 (cm/min) − *n* = 500 (RPM).

In this section, the thermal results obtained by monitoring the FSW process with IRT technique are shown. A detailed discussion about these results is present in the work of Serio *et al.* [18]. The main considerations about the thermal behavior of joints can be summarized as follows:

- The higher temperatures were measured along the retreating side for each parameter combination. In Figure 11 (Test 1), by considering three thermal profiles in orthogonal direction to the weld in a fixed time instant, it is clearly evident that temperatures on the retreating side are higher than on the advancing side during the test.
- The maximum temperature reached during the process, pixel by pixel, can be used to monitor the stationary nature of the process. Figure 12 shows the Tmax maps obtained analyzing the sequences of Test R2T1 and Test R2T4, recorded with the Flir sc 640 thermal camera. These maps represent the maximum temperature reached by each pixel during the test independently of time [17,18]. A non-uniform distribution of Tmax along the welding direction is clearly evident above all in Test R2T4. These maps confirm that the stationary conditions of the welding process along the joints have not yet been reached.
- The maximum heating slope (MSHC) of thermal profiles evaluated on the surface of joints can be used for monitoring the process parameters. This parameter is more sensitive than maximum temperature, as it is directly correlated with the energy and then the heat supplied during the welding process.

**Figure 13:**
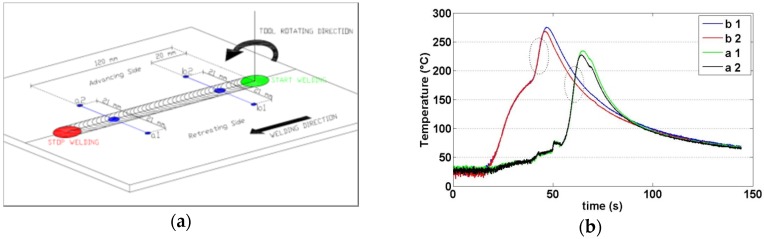
(**a**) Positioning of the measurement points along the welding line, used for the analysis of MSHC; (**b**) Thermal profile obtained for points a1, a2, b1 and b2 (Test R1T3).

The slope of the heating curve was evaluated in two sections of the work piece, both on the retreating and advancing side (Figure 13) in order to obtain a total of four values for each test. The considered points are positioned at 120 mm from section A (a1 and a2 in Figure 5) and at 20 mm from section A (b1 and b2 in Figure 5), characterized by the transience phase of the process due to the penetration of the tool. In particular, for each thermal profile of each point, the maximum slope of heating curve was evaluated (MSHC).

The MSHC values were evaluated through the processing of thermographic data. In particular, the linear best fit of 70 temperature data of each profile (Figure 13a) was used to assess the maximum slope value. Figure 13b shows the thermal profiles obtained during Test R1T3 and the range of temperature data used for evaluating MSHC value.

Table 4 shows the MSHC values assessed for each test expressed in terms of angular measurement (degrees) compared to the horizontal axis.

A statistical analysis (ANOVA) has been carried out in order to verify the statistical significance of the effect produced by process parameters on MSHC [18]. ANOVA is a statistical instrument, developed in order to verify the significance of the differences between the arithmetic means of two or more similar statistical populations [35].

The analysis of ANOVA showed that the parameter MSHC is influenced both by tool rotation speed n and traverse speed *v* (more details about the ANOVA analysis are present in the work [18]). The dependence on position *p* is due to the non-stationary condition of the process. Finally, an empirical model (Equation (1)) can be obtained in order to correlate all the significant parameters.

MSHC = 83.558 + 0.0722*v* + 0.002668*n* + 0.00688*p* (1)

### 3.4. Tensile Behavior of Joints

Specimens were cut distant to the initial and the final section (where tool exit was located) of the weld because around these sections the weld process is not stationary. In particular, they were machined according to standard UNI EN ISO 6892-1:2009 [33] and they were obtained in orthogonal direction with respect to the rolling direction. The gauge section of specimens was located within the welded zone and the geometrical dimensions chosen were 12 mm width and 200 mm length for a gauge length of 50 mm.

#### 3.4.1. Statistical Analysis on the Tensile Results

**Figure 14:**
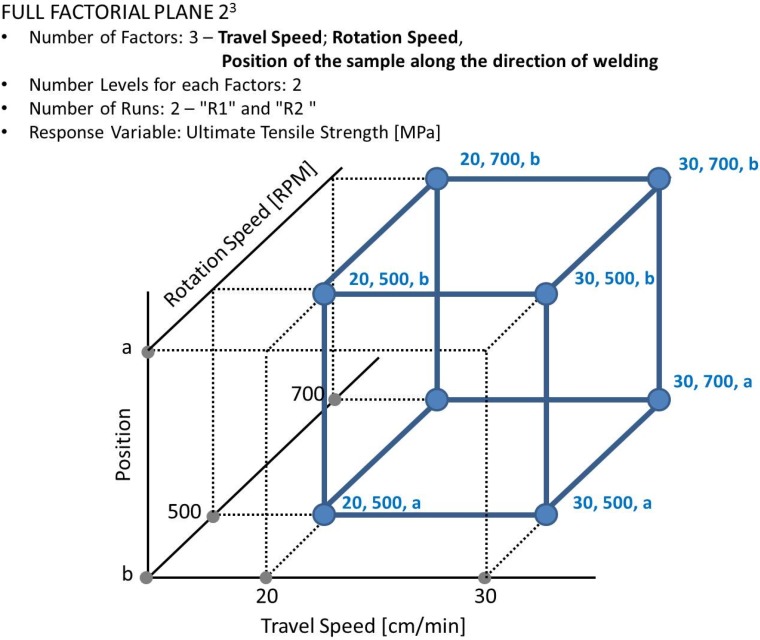
2^3^ experimental design.

An ANOVA was carried out considering a 23 full factorial experimental plan, as shown in Figure 14, in order to study the effects of each considered parameter on UTS of welded joints, with a significance level of 0.05. The considered factors were: the rotation speed, the traverse speed and the position of specimen along the welds.

**Figure 15:**
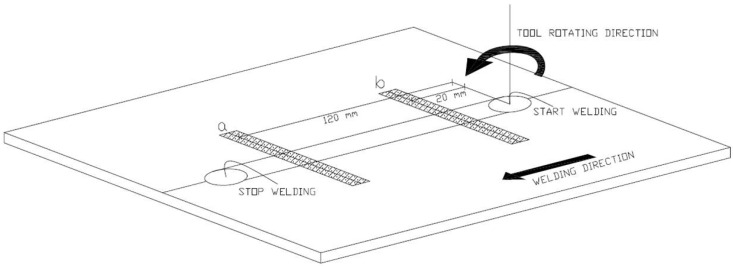
Positioning of the two samples a and b, along the welding direction, used for ANOVA.

In the statistical analysis, two specimens were considered from each welded joint, as shown in Figure 15: specimen a, placed at 120 mm from section A, and specimen b placed at 20 mm from section A.

UTS values used for ANOVA are shown in the Table 5.

**Figure 16:**
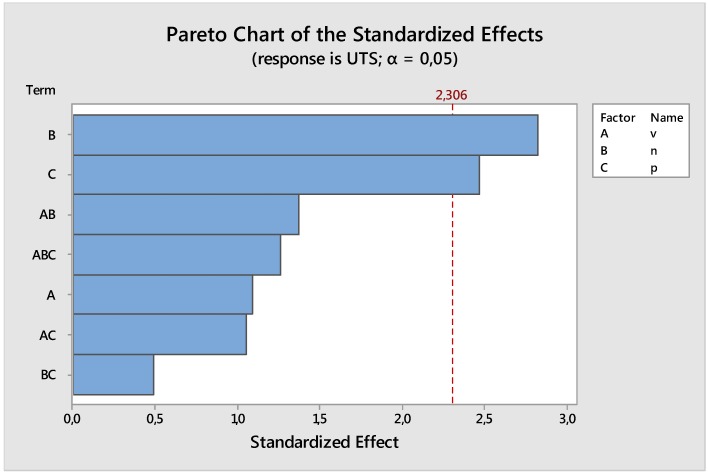
Pareto Graph for the analysis of variance for UTS *versus* traverse speed, rotation speed, and position of samples along the welding direction.

The statistical analysis, performed with the software MINITAB, shows a significant influence of the factors rotation speed and position on the UTS. The main results of this analysis are indicated and summarized in Table 6 and in the Pareto Graph (Figure 16).

The ANOVA confirms variations of the mechanical properties of joints along the weld due to the non-stationary nature of FSW process (statistical significance of position).

Moreover, there is no important significance of the rotation speed while the interactions between factors are not statistically significant (see results in Table 6 and in the Pareto graph (Figure 16).

A second ANOVA (Table 7) was carried out without taking into account the traverse speed in order to obtain four replicates for each test. The results confirmed the statistical significance of the rotation speed and position while the interaction is still insignificant.

Finally, an empirical model was assessed (Table 8) in order to predict the UTS values along the weld for a given rotation speed value and position:

UTS = 174 − 0.180*n* + 0.315*p* (2)

#### 3.4.2. Correlation between Tensile and Thermal Properties

Previous statistical analysis showed the dependence of UTS and maximum heating slope (MSHC) from the rotation speed of tool and position along the welding direction. Thus, it was possible to assess an empirical model in order to estimate the UTS from the MSHC.

**Figure 17:**
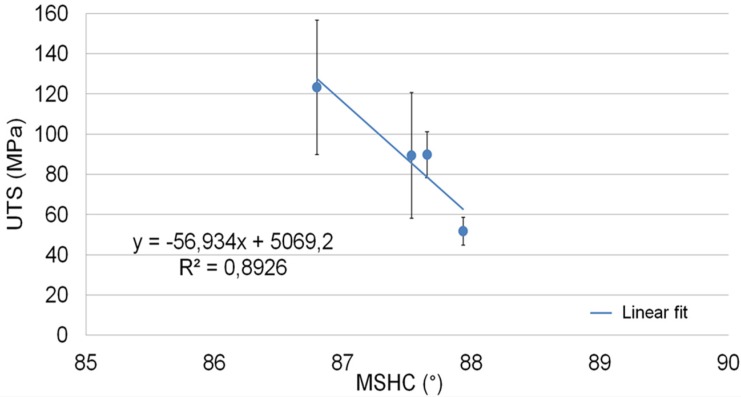
Linear trend of UTS *vs.* MSHC and error bands.

In this model, the traverse speed of tool and the side of welding were not considered as significant and thus, as replication data. In this way, eight values of UTS and MSHC were obtained for each value couple (*n*, *p*). Table 9 shows the average and standard deviation value for each series of considered data. These values were used to obtain the empirical model between UTS and MSHC (Figure 17).

Figure 17 shows the equation of the model obtained with a regression analysis and the error bands in terms of standard deviation along the *y*-axis. This model allows us to evaluate the quality of welded joints in terms of UTS, monitoring the thermal behavior of material during a non-stationary FSW process. Elevated values of standard deviation are probably due to the non-stationary condition of FSW process. Moreover, other errors could be due to misalignments between the points used to assess the MSHC values and the position of specimens used for the tensile tests.

## 4. Conclusions

In this work, the mechanical and thermal behavior of 5754-H111 plates joined by Friction Stir Welding was studied by means of destructive and non-destructive tests. The main results can be summarized as follows:

- The macrographs and the visual inspections revealed a good mixing and a good penetration of the tool in the joints, except for the joints section realized using the highest rotation speed of tool (*n* = 700 RPM; tests: R2T3, R1T2 R2T2), where they revealed defects such as cavity, due to inappropriate contribution of heat input and stirring rate.
- In order to evaluate the effects of process parameters on the quality of joints, tensile tests were carried out on specimens obtained according to the standards. In particular, a statistical analysis (ANOVA) showed that the mechanical strength of joints (UTS) is influenced by the tool rotation speed. Moreover, different values of UTS were obtained along the weld direction due to non-stationary conditions of the process.
- The potentiality of thermography for the on-line monitoring of the FSW process was demonstrated along with the possibility to evaluate the quality of joints in terms of ultimate tensile strength (UTS) by monitoring a thermal parameter (maximum heating slope of thermal profiles evaluated on surface of joints).

## Figures and Tables

**Table 1:** Chemical composition of the 5754-H111 aluminum alloy.

Alloy	Si	Fe	Cu	Mn	Mg	Cr	Ni	Zn	Ti	Other
AA5754-H111	0.40	0.40	0.10	0.50	2.60–3.60	0.30	0.05	0.20	0.15	0.05

**Table 2:** Mechanical properties of AA5754-H111 perpendicular to the rolling direction.

*R*_m_ (MPa)	*R*_p_ (0.2) (MPa)	HB	*E* (MPa)	Density (g/cm^3^)	Thermal Conductivity (W/m°C)	Specific Heat (Cal/kg°C)
190	80	53	70,000	2.65	138	0.213

**Table 3:** Summary of results obtained by the visual inspection.

			Traverse Speed (cm/min)					
			20			30		
	**Rotation Speed (RPM)**	**500**	**R1T1**	Weld Pitch	**R2T1**	**R1T4**	Weld Pitch	**R2T4**
**Internal void**			absent	0.40	absent	absent	0.60	absent
**Surface defect**			absent		absent	absent		absent
**Lack of penetration**			absent		absent	absent		absent
**Excessive flash**			absent		absent	absent		absent
		**700**	**R1T3**	Weld Pitch	**R2T3**	**R1T2**	Weld Pitch	**R2T2**
**Internal void**			absent	0.29	present Type: “tunnel”	present Type: “tunnel”	0.43	present Type: “tunnel”
**Surface defect**			absent		present	present probably formed during the transitional phase		present probably formed during the transitional phase
**Lack of penetration**			absent		absent	absent		absent
**Excessive flash**			absent		present on Advancing Side	present on both sides of the joint		present formed more on Advancing Side

**Table 4:** Values of MSHC for each test.

		Traverse Speed (cm/min)							
		20				30			
**Rotation Speed (RPM)**	**500**	**R1T1**		**R2T1**		**R1T4**		**R2T4**	
		**a1:**	87.246°	**a1:**	87.587°	**a1:**	87.968°	**a1:**	87.575°
		**a2:**	86.800°	**a2:**	87.433°	**a2:**	87.911°	**a2:**	88.448°
		**b1:**	86.051°	**b1:**	86.596°	**b1:**	89.066°	**b1:**	86.883°
		**b2:**	85.825°	**b2:**	86.161°	**b2:**	86.846°	**b2:**	87.229°
	**700**	**R1T2**		**R2T2**		**R1T3**		**R2T3**	
		**a1:**	87.229°	**a1:**	87.480°	**a1:**	88.140°	**a1:**	88.530°
		**a2:**	87.911°	**a2:**	87.735°	**a2:**	88.146°	**a2:**	88.319°
		**b1:**	87.117°	**b1:**	86.528°	**b1:**	87.371°	**b1:**	87.735°
		**b2:**	87.921°	**b2:**	87.998°	**b2:**	87.371°	**b2:**	88.228°

**Table 5:** Summary of the UTS values obtained for each welded joint for the specimens a and b.

		Traverse Speed (cm/min)							
		20				30			
**Rotation Speed (RPM)**	**500**	**R1T1**		**R2T1**		**R1T4**		**R2T4**	
		UTS Position a:	166.69 (MPa)	UTS Position a:	132.43 (MPa)	UTS Position a:	80.05 (MPa)	UTS Position a:	113.98 (MPa)
		UTS Position b:	90.66 (MPa)	UTS Position b:	99.06 (MPa)	UTS Position b:	71.99 (MPa)	UTS Position b:	97.43 (MPa)
	**700**	**R1T2**		**R2T2**		**R1T3**		**R1T3**	
		UTS Position a:	120.75 (MPa)	UTS Position a:	51.86 (MPa)	UTS Position a:	70.25 (MPa)	UTS Position a:	114.87 (MPa)
		UTS Position b:	56.06 (MPa)	UTS Position b:	46.55 (MPa)	UTS Position b:	44.29 (MPa)	UTS Position b:	59.95 (MPa)

**Table 6:** General Linear Model: UTS *versus* traverse speed, rotation speed, and position of samples along the welding direction.

Factor		Levels	Values			
1	Traverse Speed – *v* (cm/min)	2	20; 30			
2	Rotation Speed – *n* (RPM)	2	500; 700			
3	Position – *p* (mm)	2	20; 120			
**Analysis of variance for UTS, using Adjusted SS for Tests**						
**Source**		**DF**	**Adj SS**	**Adj MS**	***F*-Value**	***P*-Value**
Model		7	13,034.4	1862.1	2.87	0.081
Linear		3	9909.5	3303.2	5.09	0.029
*v*		1	773.5	773.5	1.19	0.301
*n*		1	5173.6	5173.6	7.97	0.022
*p*		1	3962.4	3962.4	6.11	0.039
2-Way Interactions		3	2088.5	696.2	1.07	0.414
*v* × *n*		1	1216.8	1216.8	1.88	0.208
*v* × *p*		1	715.7	715.7	1.10	0.324
*n* × *p*		1	156.1	156.1	0.24	0637
3-Way Interactions		1	1036.4	1036.4	1.60	0.242
*v* × *n* × *p*		1	1036.4	1036.4	1.60	0.242
Error		8	5191.0	648.9	–	–
Total		15	18,225.4	–	–	–
**Significance Level: 0.05**						

*S* = 25,4730; *R* − *S*_q_ = 71.52%; *R* − *S*_q_ (adj) = 46.60%.

**Table 7:** General Linear Model: UTS versus traverse speed, rotation speed, and position of samples along the welding direction.

Full Factorial Design								
Factor		Levels		Values				
1	Rotation Speed (RPM)	2		500; 700				
2	Position	2		(samples type) a; b				
	Base Design:	2; 4						
	Runs:	16						
	Replicates:	4						
**Two-way Anova: UTS *versus* rotation speed; position samples along the welding direction**								
**Source**			**DF**		**SS**	**MS**	***F***	***P***
Rotation Speed			1		5173.6	5173.57	6.95	0.022
Position			1		3962.4	3962.39	5.32	0.040
Interaction			1		156.1	156.06	0.21	0.655
Error			12		8933.4	744.45	–	–
Total			15		18,225.4	–	–	–
**Significance Level: 0.05**								

*S* = 27.28; *R* − *S*_q_ = 50.98%; *R* − *S*_q_ (adj) = 38.73%.

**Table 8:** Surface Plot of UTS *vs.* rotation speed (*n*) and position.

Regression Analysis: UTS *versus* *n*; Position										
**Predictor**	**Coef**			**SE Coef**		***T***			***P***	
Constant	174.41			41.26		4.23			0.001	
*n*	−0.17982			0.0661		−2.72			0.018	
position	0.3147			0.1322		2.38			0.033	
**Analysis of Variance**										
**Source**		**DF**	**SS**		**MS**			***F***		***P***
Regression		2	9136.0		4568.0			6.53		0.011
Residual Error		13	9089.4		699.2			–		–
Total		15	18,225.4				–	–		–

*S* = 26,4421; *R* − *S*_q_ = 50.1%; *R* − *S*_q_ (adj) = 42.5%.

**Table 9:** UTS and MSHC values used to obtain the empirical model shown in Figure 17.

Parameters		UTS (MPa)		MSHC (°)	
*n* (RPM)	*p* (mm)	Average	Standard Dev.	Average	Standard Dev.
500	20	89.79	87.66	11.49	0.69
700	20	51.71	87.94	6.94	0.44
500	120	123.29	86.80	33.48	0.87
700	120	89.43	87.53	31.20	0.55

## Abbreviations

The following abbreviations are used in this manuscript:

### Abbreviations

FSW: Friction Stir Welding
AA: Aluminum Alloy
UTS: Ultimate Tensile Strength
IRT: Infrared Thermography
HAZ: Heat Affected Zone
FSWP: Friction Stir Welding Process Parameters
Tp: Thermal Parameters
WP: Weld Pitch
TMAZ: Thermo-mechanically affected
MSHC: Maximum Heating Slope
